# Technical and cost analysis of zero-emission high-speed ferries: Retrofitting from diesel to green hydrogen

**DOI:** 10.1016/j.heliyon.2024.e27479

**Published:** 2024-03-07

**Authors:** Masih Mojarrad, Rebecca Jayne Thorne, Kenneth Løvold Rødseth

**Affiliations:** a Department of Physics, University of Oslo, PO Box 1048 Blindern, 0316, Oslo, Norway; b Institute of Transport Economics, Gaustadalléen 21, 0349, Oslo, Norway

**Keywords:** Compressed hydrogen gas, Liquid hydrogen, Superconducting propulsion system, Zero-emission high-speed ferry

## Abstract

This paper proposes a technical and cost analysis model to assess the change in costs of a zero-emission high-speed ferry when retrofitting from diesel to green hydrogen. Both compressed gas and liquid hydrogen are examined. Different scenarios explore energy demand, energy losses, fuel consumption, and cost-effectiveness. The methodology explores how variation in the ferry's total weight and equipment efficiency across scenarios impact results. Applied to an existing diesel high-speed ferry on one of Norway's longest routes, the study, under certain assumptions, identifies compressed hydrogen gas as the current most economical option, despite its higher energy consumption. Although the energy consumption of the compressed hydrogen ferry is slightly more than the liquid hydrogen counterpart, its operating expenses are considerably lower and comparable to the existing diesel ferry on the route. However, constructing large hydrogen liquefaction plants could reduce liquid hydrogen's cost and make it competitive with both diesel and compressed hydrogen gas. Moreover, liquid hydrogen allows the use of a superconducting motor to enhance efficiency. Operating the ferry with liquid hydrogen and a superconducting motor, besides its technical advantages, offers promising economic viability in the future, comparable to diesel and compressed hydrogen gas options. Reducing the ferry's speed and optimizing equipment improves fuel efficiency and economic viability. This research provides valuable insights into sustainable, zero-emission high-speed ferries powered by green hydrogen.

## Highlights

• Technical and cost analysis of a diesel ferry retrofitted with hydrogen technology.
• CH_2_ can efficiently and economically power a ferry, lowering OPEX.
• Varying operation speed impacts energy use, allowing further OPEX reduction.
• LH_2_ enables superconducting motor use, enhancing efficiency and reducing energy-loss.
• LH_2_ not currently cost-competitive but is expected viable with higher production.

## 1 Introduction

The maritime industry, despite all improvements in efficiency and greenhouse gas (GHG) emissions reduction per unit, remains one of the primary sources of carbon emissions globally [1,2]. Among these emissions, high-speed ferries contribute significantly, accounting for one of the highest portions of pollutants per passenger-kilometer [3,4]. According to the Paris agreement in 2015, GHG emissions from the maritime industry need to be reduced by at least 50% by 2050 compared with 2008 emissions [5], with a new ambitious target under the revised IMO GHG strategy to reach net-zero GHG emissions from international shipping by 2050 [6]. Norway is a European leader in electromobility uptake and is at the forefront of efforts to decarbonize transportation, especially in the marine sector. Among key measures, the Norwegian government has set requirements and restrictions for maritime transportation (including high-speed ferries), necessitating that these must transition to zero-emission propulsion by 2025 [7]. Currently, diesel represents 95% of marine fuels used. Alternative options, such as green hydrogen—comprising compressed hydrogen (CH_2_) and liquid hydrogen (LH_2_)—combined with Proton Exchange Membrane (PEM) fuel cells, hold promise for providing zero-emission power as a replacement for diesel [[8], [9], [10], [11], [12], [13]].

PEM fuel cells utilizing hydrogen are viable alternatives to conventional diesel engines due to their higher efficiency, reduced noise, and absence of vibration and pollutants. However, their widespread adoption in maritime industries faces challenges, notably the high costs associated with PEM fuel cells and the substantial requirement for pure hydrogen as a feed. Despite these obstacles, advancements in technology and the scaling up of PEM fuel cell production could contribute to increasing cost-effectiveness [14]. As an example, fuel cell system prices have decreased by one-sixth from 2005 to 2019, showcasing the positive impact of technological developments [15]. Moreover, if LH_2_ is used as the feed, there would be low contamination in PEM fuel cells since the liquefaction of hydrogen extracts impurities [16].

**Fig. 1:**
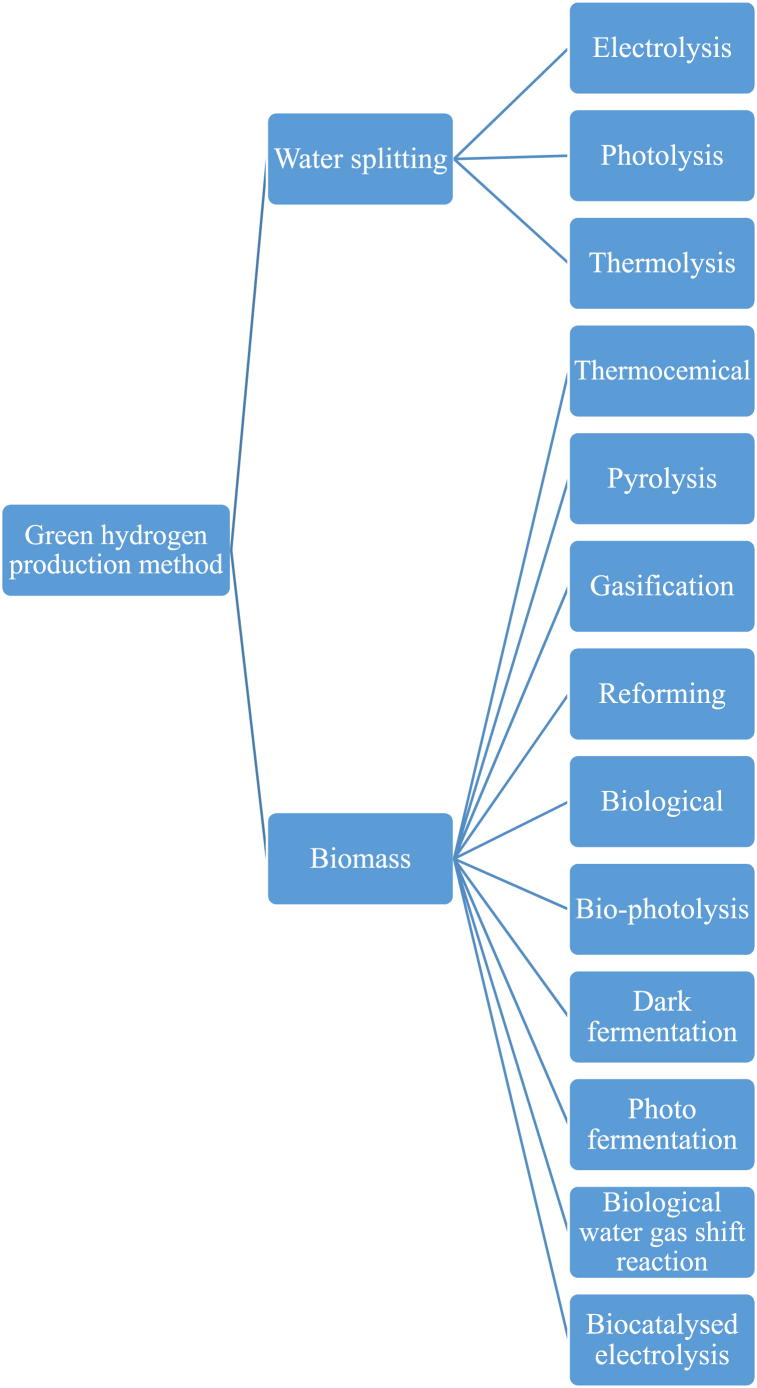
Different green hydrogen production approach (adapted from Ref. [18])

Hydrogen is characterised by thirteen distinct colours depending on its production approach. Within this spectrum, green hydrogen commits to exclusive utilization of renewable sources, predominantly water, coupled with energy inputs derived from sustainable means such as solar and wind power [17,18]. Two production technologies dominate (Fig. 1), with several approaches for each (explicitly described in Refs. [18,19]). The first technology is water splitting through electrolysis, a process that involves breaking the chemical bonds between hydrogen and oxygen in water to release hydrogen gas. The second technology involves the use of biomass, which includes a variety of plant and animal-based materials such as remnants of energy crops, forest residues, industrial by-products, and organic waste. These biomass sources represent a renewable reservoir of energy that can be converted into hydrogen. Green hydrogen production and use is increasingly driven by policy which can be attributed to its renewability, reduced lifecycle GHG emissions, and minimal sulfur content. It is also noteworthy that an increasing number of technologies are being developed for green hydrogen production, as described in references such as [[20], [21], [22], [23]]. Given its promise for environmentally sustainable energy production and the rapid advancement in technological maturity, this article specifically focuses on the application of green hydrogen in ferry transportation.

Use of green hydrogen in high-speed ferries potentially conveys many benefits. Most notably, the potential exists to greatly reduce the lifecycle GHG emissions, with one study estimating a GHG reduction of 91% on a well-to-wake basis [24]. As with other types of hydrogen, use in high-speed ferries is considered as safe as conventional ferries under normal operation conditions [25,26]. Nevertheless, challenges remain, particularly in economic terms due to high green hydrogen production costs, which several feasibility studies examining the adoption of hydrogen in maritime industries identify as a significant adoption barrier when compared to the lower cost of fossil fuels [[27], [28], [29]]. In contrast, grey hydrogen, produced from natural gas, costs about 50% less than green hydrogen but results in considerably higher lifecycle GHG emissions [30]. Several studies have performed techno-economic analysis to compare the overall operating expense (OPEX) of hydrogen-powered maritime vehicles with diesel counterparts. Whilst one [31] suggests that selecting an appropriate hydrogen storage (CH_2_) and fuel cell system for the boat could result in reduced OPEX compared with a diesel internal combustion engine system, another [32] indicates that CH_2_ still struggles to compete with diesel in terms of price in the maritime industry, primarily due to the high cost of hydrogen fuel production. It appears that the outcomes of these comparisons may vary depending on the specific case study considered. In the case of LH_2_ ferries, there is more agreement among the literature, with some indications the OPEX of an LH_2_ ferry is higher than that of a diesel-fueled ferry [24,33]. However, it is anticipated that the cost of green hydrogen, especially in liquid form (LH_2_), will decrease in the future with expanding production volumes, making it more competitive with conventional fuels such as diesel [3]. Table 1 illustrates some results from techno-economic analysis of various hydrogen vessels, as discussed in selected articles.

**Table 1:** Comparison of techno-economic analysis results for hydrogen ferries in various studies.

Articles	Evaluation of the feasibility of replacing fossil-fuel ferries with hydrogen counterparts
Feasibility of the Zero-V: A zero-emissions hydrogen fuel-cell coastal research vessel [24]	The capital cost of constructing hydrogen vessels is comparable to other types of ferries. However, the operating and maintenance costs of LH_2_ vessels are 7.7% higher than those of diesel vessels when LH_2_ is produced from fossil-fuel resources.
Designing, sizing and economic feasibility of a green hydrogen supply chain for maritime transportation [39]	While the current price of hydrogen is too high in comparison with diesel, it could become a viable alternative for maritime fuel if the hydrogen price decreases to the breakeven point of $5. Factors such as an increase in oil prices, the implementation of CO_2_ tax policies, and a decrease in electricity prices could contribute to making hydrogen more competitive with diesel in the near future.
Techno-economic analysis of green hydrogen ferries with a floating photovoltaic based marine fueling station [40]	Hydrogen ferries could achieve both technical and economic competitiveness with fossil-fuel combustion-based propulsion systems through the integration of floating photovoltaic systems for hydrogen production and fueling stations.
Techno-economic analysis of a decarbonized shipping sector: Technology suggestions for a fleet in 2030 and 2040 [41]	Hydrogen-based ferries have the potential to replace their fossil-fuel counterparts until 2030, provided that fuel cells follow their anticipated development trajectory and appropriate CO_2_ costs are established. Even without the incorporation of CO_2_ costs, these ferries could remain competitive until 2040, driven by a reduction in the production cost of fuel cells.
Techno-economic assessment of alternative marine fuels for inland shipping in Croatia [42]	Replacement of diesel ferries with hydrogen ones is feasible only if the cost of hydrogen production is below 3.3 $/kg.
Techno-economic assessment of advanced fuels and propulsion systems in future fossil-free ships [43]	LH_2_ is too expensive to be a viable alternative to fossil fuels in conventional ships.
Techno-economic analysis of renewable fuels for ships carrying bulk cargo in Europe [44]	Hydrogen ferries are more expensive than diesel ferries. The difference in cost analysis varies considerably in different investigations, depending on assumptions related to the capital expenses (CAPEX) of fuel production assets and fuel infrastructure costs.
Bridging the Maritime-Hydrogen Cost-Gap: Real options analysis of policy alternatives [45]	Green LH_2_ could be an economically viable investment for use as an alternative marine fuel if a carbon tax of at least 28% is imposed on conventional fossil-fuel ships; however, the infrastructure cost is not considered in this analysis.
Techno-Economic Analysis of Hydrogen for Coastal Maritime Transport Electrification [46]	Although green hydrogen offers environmental advantages, the current cost is high. Nevertheless, it could become competitive with diesel by 2050, with the estimated cost of hydrogen reaching $3/kg.

A potential reduction in the costs of liquefied hydrogen production could pave the way for the introduction of superconductivity in the maritime industry. Superconductors are materials that can conduct electricity with zero resistance, offering the possibility to replace conventional electrical devices for more efficient energy transmission. However, their practical use has been limited due to a significant drawback: they require extremely low operating temperatures to maintain their superconductive properties, making their use expensive and challenging on an industrial scale. The relevance of LH_2_ in this context lies in its low boiling point of 20 K (−253 °C), which is well within the temperature range required for superconductivity. If LH_2_ is integrated into marine technology, it can serve a dual purpose: acting as a coolant to maintain the low temperatures necessary for superconductors to function, and as a fuel for the vessel. This dual functionality could lead to a synergistic effect, where the use of LH_2_ not only reduces energy losses associated with superconductivity but also brings down the overall costs for implementing both technologies in maritime applications [34]. Reference [35] conducted an analysis on an 8-h high-speed ferry operation powered by hydrogen, and examined the impact of using superconducting motors in conjunction with LH_2_ as the primary fuel source. The findings indicated that CH_2_ proved to be the most economical option, while LH_2_ combined with superconducting motors emerged as the most efficient from a technical perspective. Nevertheless, this study did not include a comparison with a fossil fuel-powered ferry.

Rigorous policies such as those in Norway can motivate industries to adopt zero-emission energy sources, including in the marine sector. Accordingly, this paper aims to outline a viable pathway for retrofitting a diesel ferry to a green hydrogen ferry (CH_2_ or LH_2_), offering improved technical and economical functionality. Our investigation focuses on a diesel high-speed ferry named Elsa Laula Renberg, which currently operates between Bodø and Sandnessjøen. Batteries are not considered in this article since the journey distance is about 200 km and is considered a long-range distance in Norway; while batteries have already been successfully implemented for short-range passenger ferries (e.g. MS Medstraum [36]), range limitations mean that hydrogen may be more suitable for longer distances [[9], [37]]. Presenting LH_2_ as an option allows us to consider the feasibility of implementing the superconducting propulsion system, which aims to enhance the economic viability and narrow the cost gap with alternative fuels in the industry – an aspect rarely addressed in existing literature. This initiative not only contributes to environmental carbon emission reduction but also results in cost savings. Overall, this paper presents a technical and cost analysis to assess how transitioning to a zero-emission system would impact costs, energy demands, and energy losses. It also aims to determine the optimal green hydrogen replacement option for diesel fuel, which could also be helpful for similar investigations. Moreover, drawing inspiration from Havre et al. [38], who proposed speed optimization for achieving the most efficient abatement cost, we analyze the potential impact of varying the ferry's operational speed on both the technical and economic facets of our case study.

## 2 Methodology

**Fig. 2:**
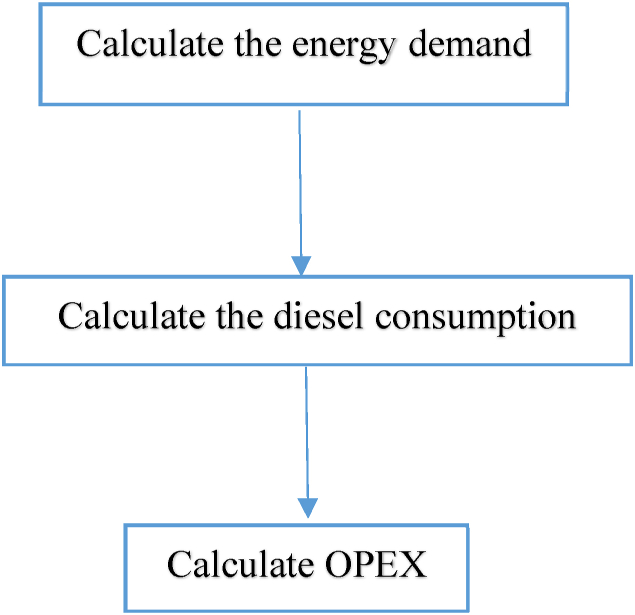
Overview of the energy and OPEX calculation methodology for the diesel ferry.

**Fig. 3:**
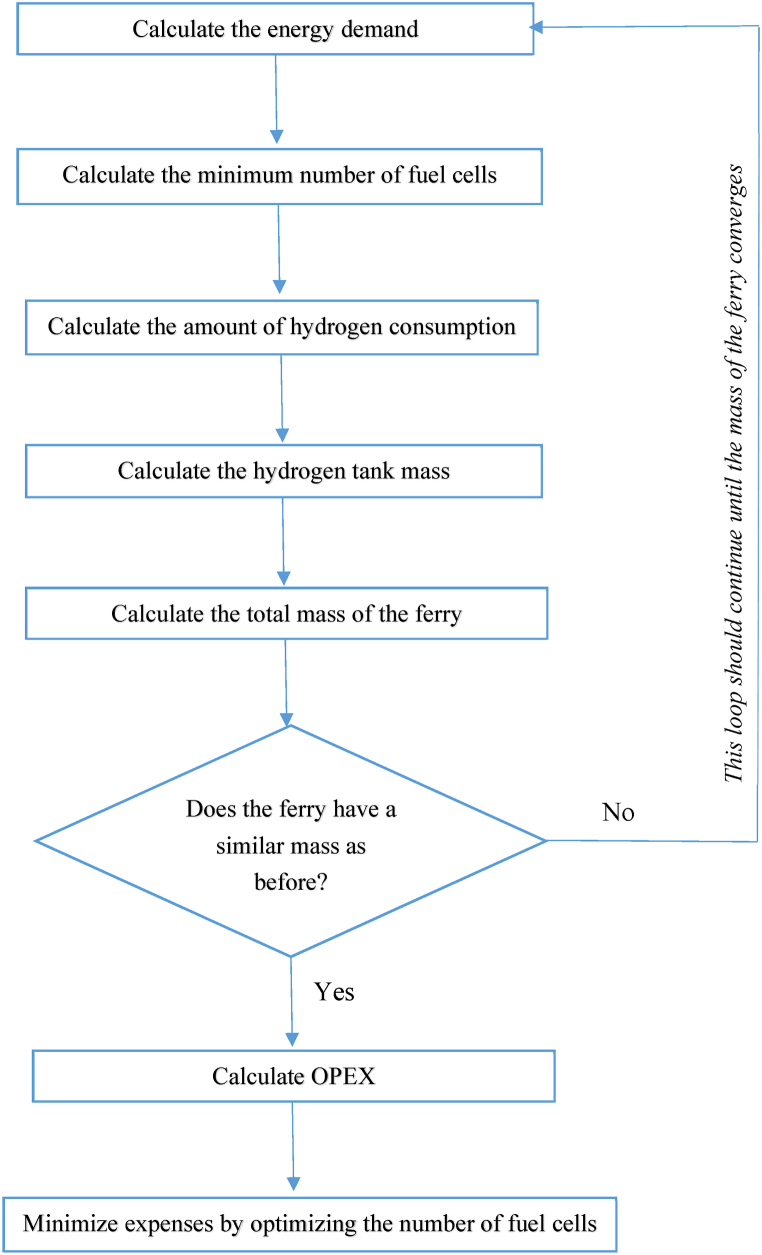
An overview of the methodology for the iterative calculation of energy and OPEX for the (retrofitted) hydrogen ferry.

This section provides an overview of the methodology used in the technical and cost analysis model. The model framework developed herein draws on Ref [47], which describes the retrofitting of a diesel ferry to a hydrogen ferry, and [35], which outlines the procedure for determining the operating expense (OPEX) of the hydrogen ferry. This study combines and modifies these methodologies to create a new approach, and therefore all equations in this section are adapted to align with the desired approach. To establish a benchmark, the model begins by obtaining the OPEX for the diesel ferry, as shown in Fig. 2. The subsequent step, illustrated in Fig. 3, involves retrofitting the diesel ferry to a hydrogen ferry and re-calculating the OPEX.

**Fig. 4:**
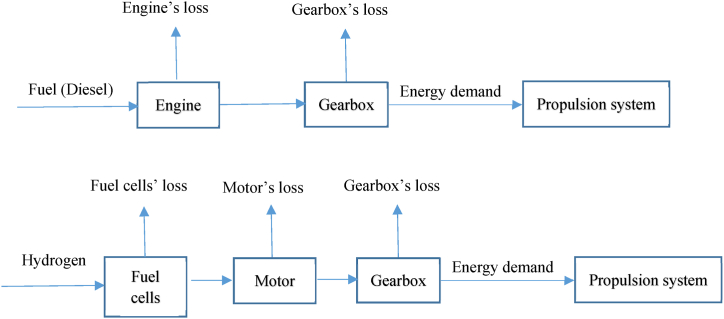
The schematics of the operation of the ferry – top: diesel ferry; bottom: hydrogen ferry.

Understanding of the diesel and hydrogen propulsion systems, including energy distribution and losses, can be gained from Fig. 4. Although there are similarities in operation, there are also notable differences such as the removal of the engine and the inclusion of motors combined with fuel cells in the system when retrofitting. The efficiency of the diesel engine is considered constant, but the efficiencies of the fuel cells and motor change respect to the power load. Moreover, some other pieces of equipment are also replaced. When conducting the calculations, the influence of the mass of the equipment is taken into consideration. This aspect will be elaborated on in subsequent sections. Each step of flowcharts shown in Fig. 2, Fig. 3 is described in the following subsections.

### 2.1 Energy demand

To calculate the energy demand, one needs to extract the load profile of the ferry. The load profile shows the change in the power demand during the operational time. Therefore, the energy demand is obtained by Equation (1).

(1) $$Energy\;demand\;\left[kJ\right]=\sum_{power=0}^{\begin{array}{c} maximum\\ power\;demand \end{array}}power\;demand\;\left[kW\right]\times {time}_{power\;demand}\;\left[s\right]$$

Since the diesel ferry serves as the benchmark, the energy demand obtained in equation (1) pertains to the diesel ferry. To calculate the energy demand of the hydrogen ferry, the same method as the diesel ferry is utilized, as described by Equation (1) with some adjustments in the power demand. The power demand value is dependent on both the speed and the mass of the ferry. Although the hydrogen ferry is assumed to operate at the same speed as the diesel ferry, the mass of the ferries differs due to the implementation of different equipment, which impacts the power demand and, in consequence, the energy demand.

As an initial approximation, it is assumed that the mass of the hydrogen ferry and diesel ferry are identical. Therefore, the initial estimate for the energy demand of the hydrogen ferry is set equal to the energy demand of the diesel ferry. In other words, the first step of Fig. 3, at the beginning of the iterative approach, is equal to the first step of Fig. 2, which calculates the energy demand of the diesel ferry. In the subsequent steps, the impact of mass on the energy demand is taken into account for the hydrogen ferry, and the energy demand value is adjusted iteratively until it converges to a certain value.

Through this iterative process, the influence of the mass on the energy demand is gradually accounted for, resulting in a more accurate estimation of the energy requirements for the hydrogen ferry. In each iterative process, the energy demand could be calculated by Equation (1), while the power demand modifies in each iteration. The method of obtaining power demand for each iteration is later discussed in section 2.15.

#### 2.1.1 Fuel mass requirement

As shown in Fig. 4, not all the energy content of the fuel is converted to the energy demand for the propulsion system, with a considerable amount of the energy lost in the engine and gearbox for the diesel ferry. The efficiencies of the gearbox and engine are assumed to be 97% and 40%, respectively [3,35]. Thus, Equation (2) calculates the energy content of the diesel that needs to be fed to the engine.

(2) $$Energy\;content\;of\;diesel\;\left[kJ\right]=\frac{Energy\;demand\;\left[kJ\right]}{Efficiency\;of\;the\;gearbox\times Efficiency\;of\;the\;engine}$$

To acquire the amount of diesel consumption, the energy content in Equation (2) should be divided by the calorific value (or higher heat value) of the diesel – 45.5 MJ/kg:

(3) $$Diesel\;consumption\;\left[kg\right]=\frac{Energy\;content\;of\;diesel\;\left[kJ\right]}{Calorific\;value\;of\;diesel\;\left[MJ/kg\right]\times 1000}$$

**Fig. 5:**
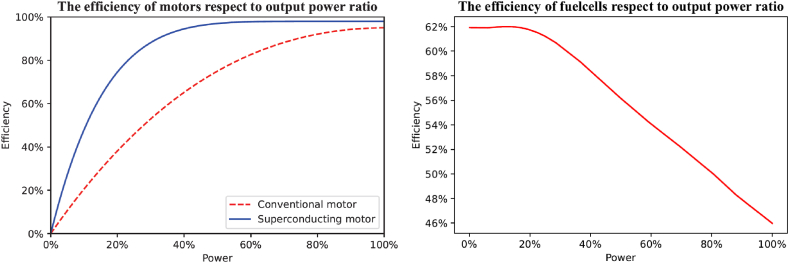
Left: The efficiency of a conventional and superconducting motor with respect to the power load (data is provided by American Superconductor Company); Right: The efficiency of fuel cells with respect to the power load (adopted from Ref. [3]).

The method of obtaining hydrogen consumption is similar to the method used for the diesel ferry. However, there is a key difference: instead of considering the efficiency of an engine, we need to take into account the efficiencies of both the motor and the fuel cells. Unlike the engine efficiency, which can be assumed to be largely constant over varying loads, the efficiencies of the motor and fuel cells vary based on their output power, which is shown in Fig. 5.

Superconductivity has a very low resistivity under a certain temperature. Therefore, Fig. 5 (left) demonstrates that the efficiency of a superconducting motor surpasses that of a conventional motor, especially at low power loads. Not only does the superconducting motor exhibit superior efficiency, but it also possesses other advantages such as being lighter, producing less noise, and having a more compact design [34]. The implementation of a superconducting motor is feasible only when utilizing LH_2_ in the ferry, as LH_2_ can serve as both fuel and coolant for the superconducting motor.

In general, both types of motors experience an increase in efficiency as the power load increases. Conversely, fuel cell efficiency exhibits an inverse relationship with power load. This can be observed on the right side of Fig. 5, where it is evident that the efficiency of fuel cells decreases as the power load rises. Therefore, the efficiency characteristics of the motors and fuel cells play a crucial role in determining the overall performance and energy consumption of the hydrogen ferry. The profile of fuel cell efficiency has an important influence on the optimization of operation expenses, which will be explained in section 2.1.7.

To obtain the energy content of hydrogen consumption, the efficiencies of the gearbox, motors, and fuel cells need to be considered because a considerable part of the energy is lost in these components. In other words, the energy content of hydrogen refers to the total amount of energy released when hydrogen is consumed as a fuel, which is obtained as follows:

(4) $$Energy\;content\;of\;hydrogen\;\left[kJ\right]=\sum_{power=0}^{\begin{array}{c} Maximum\\ power\;demand \end{array}}\frac{power\;demand\;\left[kW\right]\times {time}_{power}\;\left[s\right]}{{\;gearbox^{\prime }s\;efficiency\times motor^{\prime }s\;efficiency}_{power}\times {fuelcells^{\prime }\;efficiency\;}_{power}}$$

In relation to Equation (4), it is worth mentioning that the efficiencies of motor and fuel cells change with respect to the power demand, while the efficiency of the gearbox is assumed constant at 97 % for all ranges of power demands.

Finally, to obtain the hydrogen consumption, the amount of hydrogen energy content should be divided by the low heat value (LHV) of the hydrogen (119.96 MJ/kg):

(5) $$Hydrogen\;consumption\;\left[kg\right]=\frac{Energy\;content\;of\;hydrogen\;\left[kJ\right]}{LHV\;\left[\frac{MJ}{kg}\right]\times 1000\;\left[\frac{kJ}{MJ}\right]}$$

#### 2.1.2 Hydrogen mass inside the tank

In the previous section, fuel mass is calculated based on energy demand, but extra hydrogen needs to be stored on the ferry. For LH_2_, about 15% of the initial amount of hydrogen should remain in the tank to keep the temperature inside the tank low [29]. This implies that an additional 15% of the hydrogen consumption mass needs to be included in the tank to maintain proper cooling. For CH_2_, the hydrogen tank should have some initial pressure for refueling. Unlike most gases, the temperature of hydrogen increases when it releases at lower pressure. If the temperature of hydrogen exceeds 85 °C, it could cause some damages to the wall of the tank. Having some initial pressure inside the tank could help to control the increase of the temperature during refueling. The initial pressure of the hydrogen tank could vary depending on the mass flow rate, initial temperature of hydrogen, ambient temperature, and the final pressure of the tank [48]. However, to ensure the tank maintains sufficient pressure for refueling, an additional 8% of the initial CH_2_ consumption mass should be introduced to the 350-bar hydrogen tank [49]. Moreover, an extra amount of hydrogen needs to be considered for some unpredictable situations e.g. harsh weather. A safety margin of 30% is considered for both types of hydrogen for such situations. Therefore, the amount of hydrogen inside the tank is calculated as follows:

(6) Hydrogeninsidethetank[kg]=1.3×hydrogenconsumption[kg]×(1+refuellingmargin)

Where $refueling\;margin=\left\{ \begin{array}{l} 0.08\\ 0.15 \end{array}\right.\begin{array}{l} {\;for\;CH}_{2}\\ {\;for\;LH}_{2} \end{array}$:

#### 2.1.3 Hydrogen tank mass

Table 2 presents the gravimetric specification of the hydrogen tank concerning the amount of hydrogen it contains. The mass of the hydrogen tank can be determined by multiplying the gravimetric specification with the mass of hydrogen inside it, as indicated in Equation (7). In the case of storing hydrogen in cryogenic form, it must be heated before it can be utilized in fuel cells. Consequently, for LH_2_ ferries, an evaporator must be taken into account, and for this study, it is assumed to be an integral part of the hydrogen tank.

(7) Massofhydrogentank=Massofhydrogen×Gravimetricspecifications

**Table 2:** Gravimetric specification of different types of hydrogen tanks.

Types of tank	Gravimetric specifications (Empty tank weight/Hydrogen stored weight) [kg/kg]
350 bar compressed hydrogen tank	12.35 [50]
LH_2_ tank	8.7 [29]
LH2 tank with considering an evaporator	9.4 [29]

#### 2.1.4 Minimum required number of packs of fuel cells

One of the factors that adds mass to the ferry is the fuel cells themselves. Therefore, it is important to find out the minimum number of packs of fuel cells needed for the ferry, which can be calculated by Equation (8).

(8) $$Number\;of\;packs\;of\;fuel\;cells=\frac{Maximum\;power\;demand\;\left[kW\right]}{Maximum\;output\;power\;of\;a\;pack\;of\;fuelcells\;\left[kW\right]}$$

The maximum power demand is obtained from the load profile and corresponds to the maximum speed of the ferry. The maximum output power of the fuel cells depends on what type of fuel cells is chosen. Furthermore, the number of fuel cells used in the system is logically an integer value. Consequently, when calculating the right-hand side of Equation (8), if the result contains decimals, the mixed-integer programming should be used and the value rounded up to obtain the appropriate minimum required number of packs of fuel cells.

#### 2.1.5 The total mass of the hydrogen ferry

This study assumes that the total mass of the diesel ferry, as the benchmark, is known. The equipment used for a diesel ferry is different from a hydrogen one. Therefore, to retrofit to a hydrogen ferry, some pieces need to be removed and replaced with the appropriate equipment for the hydrogen ferry. These pieces could be different from one ferry to another. Nevertheless, some equipment usually needs to be detached: diesel engine, diesel tank, auxiliary engine, and exhaust system. Some diesel ferries might have other equipment (e.g. generators) that needs to be considered. Instead, to convert the existing ferry to a hydrogen-powered ferry, some pieces of equipment need to be added: hydrogen tank, fuel cells, DC switchboard, transformers, batteries, and drives. As explained, the equipment could be different depending on the initial facilities of the diesel ferry, and the desired transition to the hydrogen ferry.

Since the mass of the ferry is changed when it is retrofitted to a hydrogen ferry, the impact of this change on energy consumption needs to be taken into account. Reference [29] gives an approximation of how the total mass impacts the power demand: One percent change in the mass of the ferry leads to one percent change in the power demand. Therefore, the power demand can be modified using Equation (9):

(9) $$Modified\;power\;demand\;\left[kW\right]=power\;demand\;\left[kW\right]\times \frac{New\;mass\;of\;the\;ferry\;\left[kg\right]}{Former\;mass\;of\;the\;ferry\;\left[kg\right]}$$

By obtaining a new power demand, all the values from Equation (1) to Equation (9) (excluding Equations (2), (3), which are only related to the diesel ferry) should be recalculated (cf. Fig. 3). If the new obtained mass of the ferry is not similar to the former mass of the ferry, this loop should be continued until the mass of the hydrogen ferry converges to a certain value.

#### 2.1.6 Ferry's OPEX and CAPEX

Finally, OPEX is calculated by multiplying the fuel consumption by the fuel price. Based on the type of the ferry, the fuel consumption is acquired by either Equation (3) or Equation (5).

(10) $$Ferry^{\prime }s\;OPEX\;\left[\$\right]=Fuel\;consumption\;\left[kg\right]\times Fuel\;price\;\left[\$/kg\right]$$

This study exclusively calculates OPEX based on fuel consumption, omitting other expenses such as maintenance and crew salaries. According to Ref. [42], the maintenance cost of both diesel and hydrogen ferries is negligible compared with fuel costs. It is also assumed that uniform labor costs across all cases; thus, even if labor costs were included, the comparison between cases would remain the same.

Moreover, Madsen et al. [24] report that the CAPEX for constructing hydrogen ferries is comparable to that of other ferries. The cost associated with hydrogen systems constitutes only 10 percent of the total ferry cost. Consequently, the difference in CAPEX between various types of ferries is considered small.

#### 2.1.7 Minimize the expenses of the hydrogen ferry

As mentioned in 2.1.1, the profile of the fuel cell's efficiency is important for the optimization of the expenses. To be more accurate, Fig. 5 (right) indicates lower power loads result in higher efficiency. Consequently, increasing the number of packs of fuel cells improves their efficiency by reducing the power load, ultimately leading to lower hydrogen consumption. However, it is essential to consider that adding more packs of fuel cells increases the ferry's weight, resulting in higher power and energy demands. This increased energy demand implies greater hydrogen consumption if another term (the efficiency of the fuel cells) is assumed constant. Therefore, these two terms compete in optimization.

In the previous section, we calculated the OPEX based on the minimum number of fuel cell packs. To minimize the OPEX, one pack of fuel cells needs to be iteratively added and all steps recalculated while comparing the new OPEX with the previous one. This process should continue until the lowest possible OPEX is achieved. Additionally, if data on CAPEX for different components used inside the ferries and onboard facilities is available, it is advisable to minimize the sum of OPEX and CAPEX rather than just OPEX. This approach provides a more realistic assessment.

This study takes into account both OPEX and CAPEX of the fuel cells for optimization, ensuring a comprehensive analysis of the ferry's efficiency and economic considerations.

#### 2.1.8 Case study

This section introduces the specific case study under investigation and provides initial information required for the methodology.

#### 2.1.9 Economic evaluation

This article investigates retrofitting the existing high-speed ferry MS Elsa Laula Renberg as a hydrogen ferry, assuming refueling can occur at each end-stop. The vessel is a diesel high-speed ferry operating in the county of Nordland, Norway, that transports passengers from Bodø to Sandnessjøen, which is about 200 km and one of the longest routes in Norway. One ‘journey’ in this article thus equates to one trip between Bodø and Sandnessjøen. The characteristics of the ferry and its journey are given in Table 3.

**Table 3:** The characteristics of MS Elsa Laula Renberg [51].

Length	Width	Passengers	Sailing time^a^	Total mass^b^
44.2 m	10.8 m	220	5 h/journey	177.3 tonnes

a The sailing time is taken from timetables.

b The total mass involves the sum of the lightweight of the ferry, fuel, passengers, and crew. The data is provided by Paradis Nautica.

The ferry currently operates with diesel fuel, but four cases are examined in this study, as shown in Table 4. Case *a* is the current ferry operated on the route, while other cases show different possible types of hydrogen propulsion systems to retrofit the diesel one.

**Table 4:** Different cases examined in this study.

	Type of fuel	Type of propulsion system
Case *a*	Diesel	Conventional diesel engine
Case *b*	CH_2_	Conventional motor
Case *c*	LH_2_	Conventional motor
Case *d*	LH_2_	Superconducting motor

To facilitate the retrofitting of the diesel ferry to a hydrogen-powered vessel, specific equipment must be replaced, as outlined in Table 5. Additionally, the fuel price emerges as a critical factor influencing the OPEX, as detailed in Table 6. The optimization process relies on key characteristics of the fuel cells, as described in Table 7. Each of these tables is thoroughly explained in the subsequent sections for a more detailed understanding.

**Table 5:** Components of the diesel ferry that need to be replaced by the components of the hydrogen ferry [data is estimated by Paradis Nautica unless otherwise mentioned, private communication].

Diesel Ferry		Hydrogen Ferry	
**Components**^a^	**Mass [kg]**	**Components**	**Mass [kg]**
Main engines	4 × 2500	DC-switchboard and transformers	7000
Exhaust system	4 × 150	Motors^a^ (4 × 750 kW)	4400
Auxiliary engines	2 × 500	Fuel cells (400 kW) [35]	1300
Diesel tank	200	Battery (250 kW)	1500

a The mass of the conventional motor is assumed to be identical to that of the superconducting motor, including its cooling system.

**Table 6:** Range of fuel prices for different cases [35].

	Diesel	Hydrogen gas	LH_2_	LH_2, LHL_^a^
Price [$/kg]	1.4–2 [52]	2–5	9–12	2.63–7.615

a The price of LH_2_ in the future if large-scale hydrogen liquefaction plants are constructed.

**Table 7:** The characteristics of the fuel cells provided by Teco2030 [3,35].

Power [kW]	Mass [kg]	Lifetime [h]	Price [$/kW]
400	1300	25,000	1200

#### 2.1.10 Load profile of the ferry

**Fig. 6:**
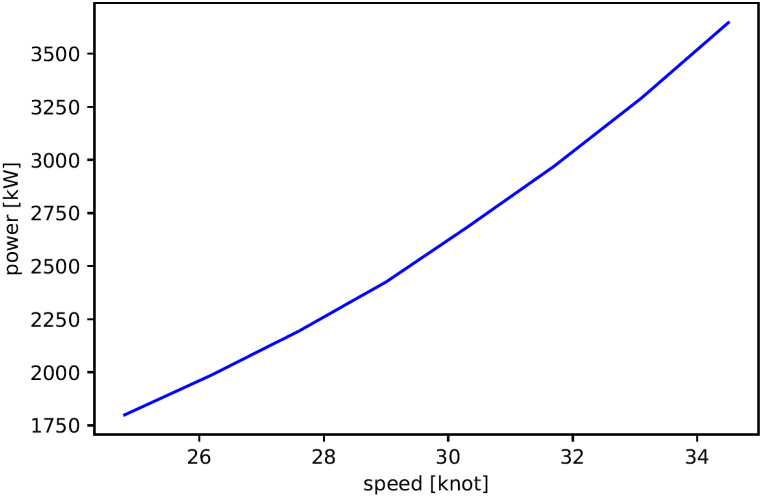
The relation between the speed and the power demand of the ferry [data is provided by Paradis Nautica].

In this study, load profiles – representing changes in power demand during the ferry's harbour-to-harbour journeys on the Bodø-Sandnessjøen route – were generated using an Automatic Identification System (AIS) model. This model relies upon use of AIS data, which is automatically transmitted from all passenger vessels providing information about location, identity, speed and course. In brief, the AIS model utilized the speed-power curve of the MS Elsa Laula Renberg obtained from Paradis Nautica (Fig. 6), and AIS data for the year 2019 for the MS Elsa Laula Renberg from the Norwegian Coastal Administration (NCA). Cleaning routines and the propulsion power demand modelling procedures were as presented in Sundvor et al. [9] For further details on the model, the reader is referred to Ref. [9] where the model was originally developed and fully presented.

Stop locations for the Norwegian passenger ferry in the year 2019, including harbour names and coordinates, as well as sequences for all routes, were sourced from the General Transit Feed Specification (GTFS). Polygons (with buffer radius 100 m) were created from the harbour data in the GFTS data, which were used to screen AIS datapoints with speed <0.4 knots to identify visited harbours for the MS Elsa Laula Renberg throughout the year. To identify movement patterns between harbours, records in the AIS dataset where SOG (Speed Over Ground) < 1.5 knots were isolated and the distance to identified harbours was calculated for the ferry. Data points where the distance to a harbour was less than a set distance (100 m) were assumed to indicate where the MS Elsa Laula Renberg was at port. This allowed the derivation of harbour visit patterns including arrival and leaving times of the ferry over the year.

By comparing harbours visited with the Bodø-Sandnessjøen route, sections of AIS data corresponding to each harbour-to-harbour path for the Bodø-Sandnessjøen route were identified. Although ferry movements vary, the path with median energy for the ferry across the year was taken as a ‘typical’ movement. Since the model identifies paths from when the MS Elsa Laula Renberg leaves a harbour to when it leaves the next harbour, median energy paths were resliced to ensure temporal and geographic coverage from zero-to-zero speed at harbours. The engine load profile with time during these paths over the complete route was then taken as representative during the journey.

**Fig. 7:**
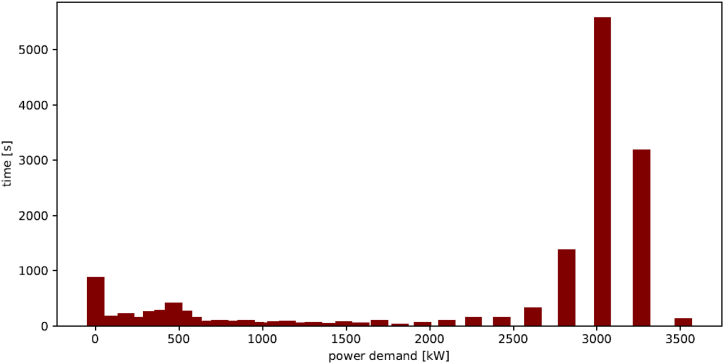
Distribution of time spent at various power demands, for the MS Elsa Laula Renberg during a trip [data from AIS modelling].

Typically, a load profile is presented as a continuous graph illustrating the power demand at each moment of the ferry's operation. The maximum power demand is obtained from the load profile and corresponds to the maximum speed for the ferry. Fig. 7 illustrates the distribution of time spent by the MS Elsa Laula Renberg at various power demands. Each power demand corresponds to a specific speed. Moreover, Fig. 7 shows that the ferry operates at higher power (or speed) levels most of the time during its operation on the route.

#### 2.1.11 Retrofitting into a hydrogen ferry

Modelling of the transition from a diesel to a hydrogen ferry in this work draws on Ref [47]. Although the operation of both ferries has similarities, differences for a retrofit relate to the removal of the diesel engine and the inclusion of motors combined with fuel cells in the system. As explained in section 2.1.5, removal and replacement of components may differ from one ferry to another. The masses of the pieces of equipment are important when adjusting the power demand of the ferry, which is shown in Table 5 for this case study. Besides the equipment mentioned in the table, the mass of hydrogen and its tank must be taken into consideration, which could vary depending on the power demand and energy loss of the ferry.

#### 2.1.12 Fuel cells

Teco2030, a Norwegian company, offers fuel cells with a maximum output power of 400 kW, weighing 1300 kg [35]. This particular fuel cell model serves as the standard reference in our study, allowing us to determine the required quantity of fuel cells to meet the energy demand and estimate the associated mass that the fuel cell packs would add to the ferry. The minimum number of fuel cells is obtained by Equation (8). In the literature, the cost of a pack of fuel cells is evaluated as $1200/kW with a lifetime of 25,000 h [3]. Therefore, the CAPEX of the fuel cells could be assessed at around $19/h.

#### 2.1.13 Propulsion system

The extracted energy from hydrogen is utilized to power the ferry's propulsion systems. The availability of LH_2_ enables the integration of a superconducting motor into the propulsion system. Unlike conventional systems that employ gearboxes to adjust motor revolutions per minute (rpm), the superconducting motor operates at the desired rpm without requiring this component. Consequently, the elimination of the gearbox results in significant energy savings.

The superconducting propulsion system, already developed by American Superconductor and deployed by the U.S. Navy, offers additional advantages. Firstly, by removing the gearbox, the mass of the propulsion system is reduced. Additionally, a superconducting motor is lighter than its conventional counterpart. However, the superconducting propulsion system necessitates a cooling system, which adds mass. In the case of large ferries, the superconducting propulsion system outweighs the conventional system in terms of mass savings. Nevertheless, for small and medium-sized ferries, including the ferry under investigation in this article, the mass of both propulsion systems is comparable [private communication with American Superconductor]. As a result, the difference in mass between the conventional and superconducting propulsion systems is disregarded in this study.

#### 2.1.14 Operating expenses of different cases

The OPEX of the ferry is obtained by Equation (10), while the fuel consumption is acquired by Equation (3) or (5) and the price of fuel is given in Table 6.

In the future, although the price of green hydrogen may fluctuate depending on the applied technology, the overall price range is expected to remain relatively stable [53,54]. Nevertheless, the high cost of hydrogen in liquid form can be attributed to the small size of LH_2_ plants. By constructing large-scale hydrogen liquefaction (LHL) plants, it becomes possible to achieve a lower cost for LH_2_ [34]. According to Ref. [55], the estimated cost of hydrogen liquefaction for large plants with a capacity exceeding 50 tonnes per day is projected to be $ 0.63–2.615/kg. This cost needs to be factored in when predicting the final cost of LH_2_ for LHL plants, in addition to the cost of hydrogen gas.

## 3 Results and discussion

In this section, we present the results and conduct a comparison between different cases. Additionally, we investigate the effect of changing the maximum speed of the ferry in different scenarios to further analyze how it impacts the energy demand, energy loss, and OPEX of the ferry.

**Fig. 8:**
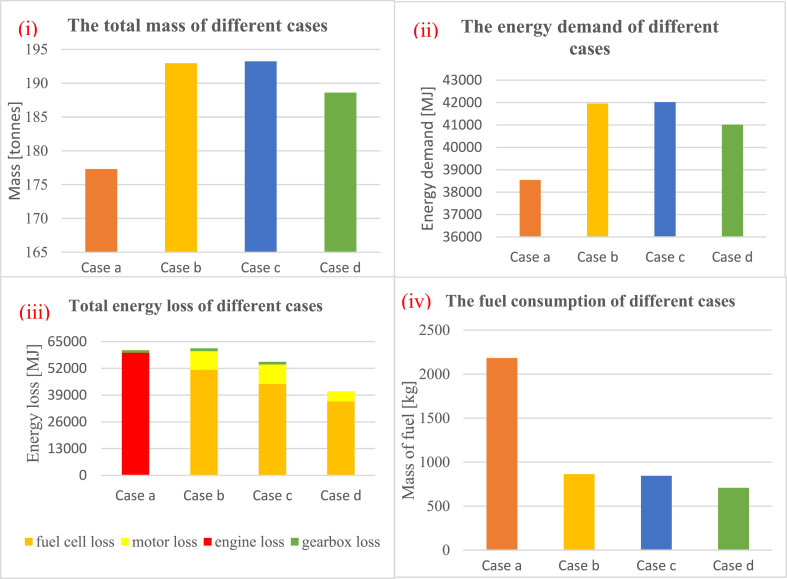
A comparison between different cases for total mass of the ferry (i), energy demand (ii), energy loss (iii), and the fuel consumption (iv).

Fig. 8-i and Fig. 8-ii show the resulting differences in the mass and energy demand of different cases. Since the components of the hydrogen ferries are heavier than the diesel ferry (cf. Table 5), the diesel ferry is the lightest option. Moreover, implementing a superconducting motor in the ferry enhances its efficiency, resulting in lower hydrogen consumption compared with the conventional motor. Consequently, the reduced hydrogen consumption allows for the use of smaller, and thus lighter, hydrogen tanks, resulting in case *d* having the lowest total mass among the hydrogen cases.

The energy demand of the different cases is directly proportional to their mass. The power demand is influenced solely by the mass of the ferry, with the assumption that all cases operate at the same speed. As a result, the heavier the ferry, the higher the power demand will be. Consequently, both the energy demand and the total mass of the ferry follow the same trend across the different cases due to their interconnected relationship.

The other factor that affects fuel consumption is the total energy loss of the ferry, which is shown in Fig. 8-iii. The total energy loss is determined by the amount of energy demand (direct proportion) and the efficiency of relevant components (inverse proportion). Although the energy demand of the diesel ferry (Case *a*) is the lowest, the low efficiency of the diesel engine makes its total energy loss comparable to that of Case *b* and Case *c*, which have higher energy demand but also higher efficiency. Fig. 8-iii also provides further insights into the energy losses associated with each equipment in different cases. In Case *a*, the dominant contributor to energy loss is the diesel engine – responsible for 98% of the total energy loss. In hydrogen-based cases (Cases *b*-*d*), the fuel cells account for over 80% of the total energy loss. This highlights the significance of optimizing fuel cells in the analysis. In contrast, the gearbox exerts minimal influence on total energy loss in all cases – contributing less than 2%. In Case *d*, where there is no gearbox, all energy loss originates from fuel cells (88%) and the superconducting motor (12%).

On the other hand, Case *d* stands out with the least amount of energy loss due to the utilization of a superconducting motor. Despite having higher energy demand compared with Case *a*, the superconducting motor exhibits remarkably high efficiency, especially at lower power loads (as evident in Fig. 5). This makes Case *d* the most efficient option in terms of energy loss, even with higher energy demand than Case *a*. The efficiency advantage of the superconducting motor makes it a favourable choice for reducing energy losses in the overall system.

Knowing both the energy demand and energy loss allows for the calculation of fuel consumption. Fig. 8-iv demonstrates that the mass of fuel required for the diesel ferry (Case *a*) is higher compared with hydrogen ferries (Cases *b*-*d*) due to the lower calorific value of diesel (45.5 MJ/kg) compared with hydrogen (120 MJ/kg). For hydrogen ferries, as they utilize the same type of fuel, the comparison of hydrogen consumption could be determined by the sum of energy demand and energy loss.

**Fig. 9:**
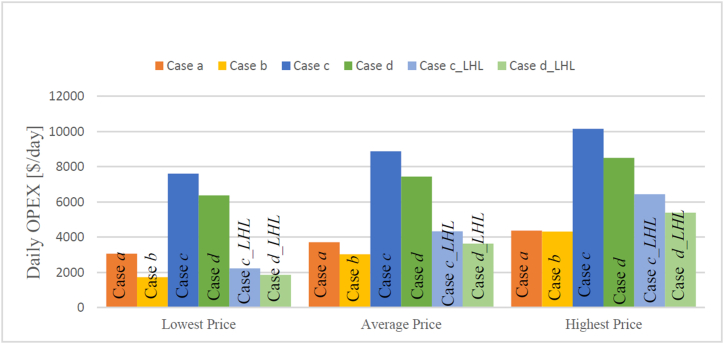
The OPEX for different cases in a journey based on the range of price.

By multiplying the fuel consumption by the respective fuel prices, the OPEX per journey is calculated and visualized in Fig. 9. The results in Fig. 9 reveal that the CH_2_ ferry (Case b) is the most economical option for this high-speed ferry. These findings are similar to Ref. [31], where a compressed hydrogen gas option is considered a promising competitor for diesel rescue boats, especially in longer mission journeys. Reference [19] also has similar findings, while in some other references [[31], [36], [37], [56]], it is believed that CH_2_ ferries will be compatible if the cost of the related materials (e.g. green hydrogen and fuel cells) are reduced. On the other hand, LH_2_ ferries (Case *c* and Case *d*) appear to be less cost-effective at present due to the higher price, in agreement also with literature studies [24,33,39]. However, it might become more competitive with diesel and CH_2_ in the future if larger LH_2_ production plants are established, leading to a decrease in LH_2_ prices. Furthermore, Fig. 9, Fig. 11 highlight that selecting LH_2_ as the fuel, along with implementing a superconducting motor, not only enhances the ferry's efficiency but also makes it more economically viable compared with conventional options.

All the parameters presented in Fig. 8, Fig. 9 are obtained after optimizing for the number of fuel cells (cf. 2.1.7). The initial minimum number of fuel cells for Case *b*, Case *c*, and Case *d* are 11, 11, and 10, respectively. However, during the optimization process aimed at minimizing expenses, the number of fuel cells changes to 11, 14, and 12 for Case *b*, Case *c*, and Case *d*, respectively. It is observed that the minimum number of fuel cells also corresponds to the optimized configuration in Case *b*. This phenomenon can be explained as follows: The addition of some packs of fuel cells improves the overall efficiency, subsequently reducing fuel consumption (and, in turn, the OPEX). However, the CAPEX of the fuel cells introduces some cost that outweighs the potential savings in the OPEX. Consequently, adding more fuel cells in Case *b* is not an optimal solution as it does not lead to the most cost-effective outcome.

### 3.1 Further analysis

The maximum speed of the investigated ferry is 34 knots, but there are some situations (such as harsh weather) or restrictions that cause the ferry does not operate at the highest possible speed. Furthermore, it helps to evaluate the optimized maximum speed for the ferry both technically and economically. In the next step, this study considers how the change in the maximum speed could affect the parameters. This change of the maximum speed is examined in two different scenarios.

#### 3.1.1 Scenario 1

In this scenario, the assumption is that all the components and equipment inside the ferry remain unchanged, but the ferry operates at a lower speed. By maintaining the same configuration and mass, the study explores the impact of running the ferry at a lower speed on various aspects including energy demand, energy loss, and OPEX. This controlled analysis helps in understanding how operational changes can affect the performance and cost-effectiveness of the high-speed ferry.

**Fig. 10:**
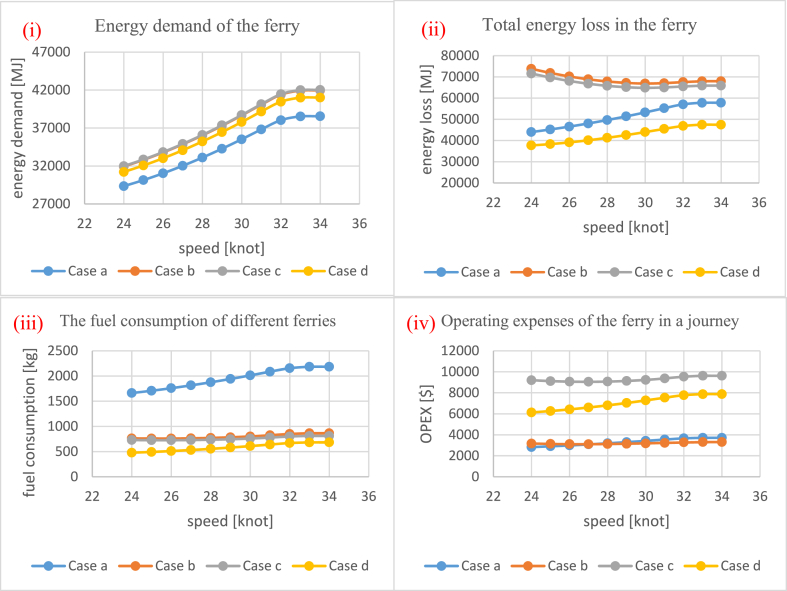
A comparison between different cases for energy demand of the ferry (i), energy loss (ii), the fuel consumption (iii), and the OPEX (iv).

Fig. 10 shows how the decrease in the maximum operational speed affects the variables. When the ferry operates at a lower speed, the operational time increases, which may cause some disutility for the passengers. Conversely, in Scenario 1, where the mass of the ferry remains constant, a decrease in the maximum speed leads to a reduction in the maximum power demand as well. As illustrated in Fig. 10-I, the decrease in power demand outweighs the increase in operational time, resulting in a decline in energy demand when the maximum operational speed is reduced.

**Fig. 11:**
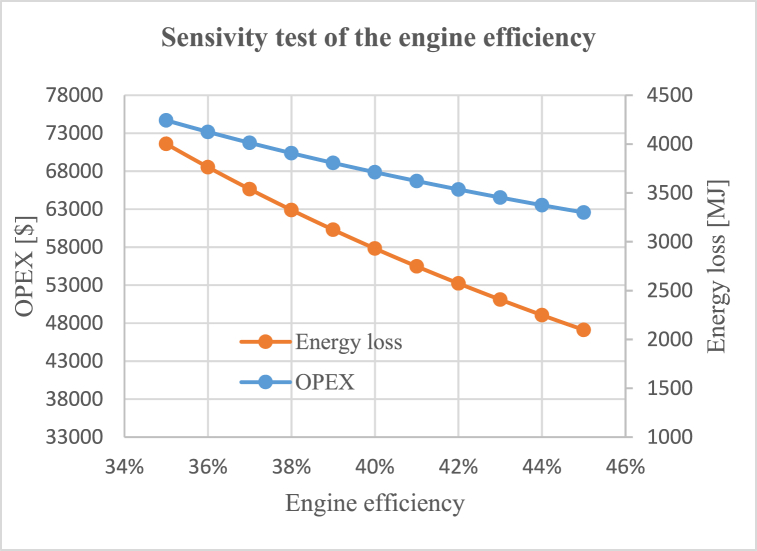
Sensitivity test of the diesel engine with respect to its average efficiency.

Fig. 10-ii describes how the maximum operational speed affects the energy loss. The mechanism for Case *a*, which is powered by diesel (cf. Fig. 4), is straightforward, as the efficiency of the engine and gearbox is assumed constant across the power range. As a result, the total energy loss of Case *a* follows a similar trend as the energy demand.

However, the mechanism for the hydrogen cases is more complex (cf. Fig. 4). The efficiency of both the fuel cells and motors is influenced by changes in the power load. When the power load decreases, there is a significant drop in the efficiency of the conventional motor, whereas the efficiency of the superconducting motor remains relatively constant for a wide range of power loads. Simultaneously, the efficiency of the fuel cells increases with the decrease in the power load.

For Case *b* and Case *c*, which operate with a conventional motor, the energy loss in the motor dominates the amount of energy saved due to the increase in fuel cell efficiency. As a result, the total energy loss for these cases still increases when the maximum operational speed decreases. However, for Case *d*, which utilizes a superconducting motor, the increase in energy loss in the motor is minimal, and the improvement in fuel cell efficiency leads to a reduction in the total energy loss when the maximum operational speed decreases.

By analyzing the trends of both energy demand and energy loss in the ferry, the trend of fuel consumption in Fig. 10-iii is understandable. In Case *a* and Case *d*, both energy demand and loss decrease with a reduction in speed. As a result, the fuel consumption for these cases follows the same decreasing trend. However, for Case *b* and Case *c*, the trends of energy demand and energy loss are opposite. In these two cases, the decrease in energy demand compensates for the increase in energy loss, leading to relatively constant fuel consumption, regardless of the maximum operational speed.

Since the OPEX follows the trends of fuel consumption, Fig. 10-iv exhibits consistency with the fuel consumption trend. However, the fuel prices in Table 6 cause the plots to shift up or down accordingly. It is important to note that the average fuel price reported in Table 6 is used to calculate the OPEX.

#### 3.1.2 Sensitivity test

In this study, the diesel engine's efficiency is assumed to be constant; however, in reality, engine efficiency varies with its power load, similar to fuel cells and motors. Ignoring this variability in engine efficiency introduces inaccuracies. To address this issue, we conducted a sensitivity test for the diesel engine to examine how the results change when considering different efficiency levels. Fig. 11 illustrates that a one percent change in the engine's efficiency results in a four percent change in energy loss and a 2.5 percent change in OPEX.

#### 3.1.3 Scenario 2

In scenario 2, the ferry operates at a lower speed permanently due to restrictions. This also allows improved optimization regarding the most appropriate maximum speed. As a result, the equipment is replaced with the most suitable components for this specific operational requirement. This includes adjustments to the amount of hydrogen, its tanks, motor, and the number of fuel cells to minimize expenses. In scenario 2, we focus solely on the hydrogen cases (Case *b*, Case *c*, and Case *d*) and compare them with the previous scenario.

**Fig. 12:**
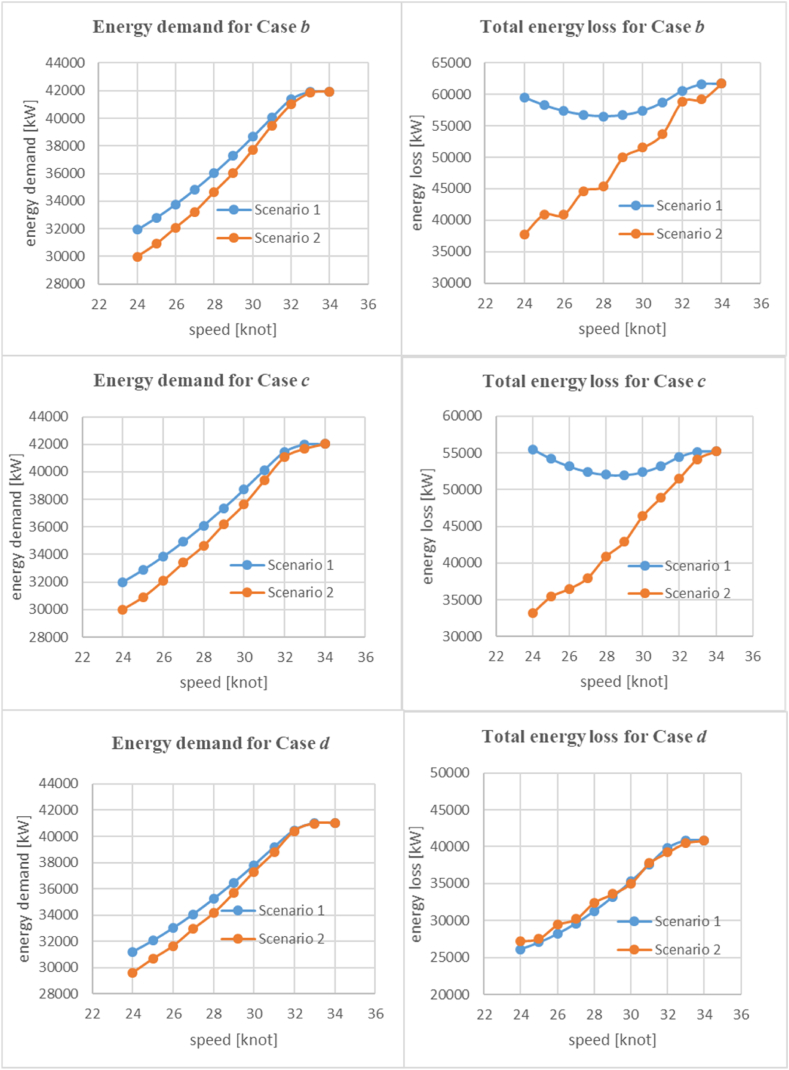
A comparison between hydrogen cases for the energy demand (left plots) and energy losses (right plots) of the ferry.

As depicted in Fig. 12, both energy demand and loss decreased in scenario 2, which is expected as the equipment is replaced with the most optimal ones to minimize expenses. However, a notable exception can be observed in the energy loss of Case *d*, which operates with a superconducting motor. In this case, it can be seen that at low speeds, the energy loss in scenario 2 is higher compared with scenario 1. It is important to note that the equipment would be replaced to achieve the least amount of expenses, which might not necessarily be optimal for other parameters. In the case of Case *d*, the energy loss for the superconducting motor remains more or less the same in both scenarios, as its efficiency is almost constant in the high range of power. However, the energy loss in fuel cells increases in scenario 2, primarily due to the lower power load resulting from the removal of some packs of fuel cells. While the total energy loss increases in scenario 2 at low speeds compared with scenario 1, it is worth mentioning that some expenses could be saved by removing some fuel cells. Furthermore, removing several fuel cells results in a lighter ferry, leading to reduced energy demand overall.

**Fig. 13:**
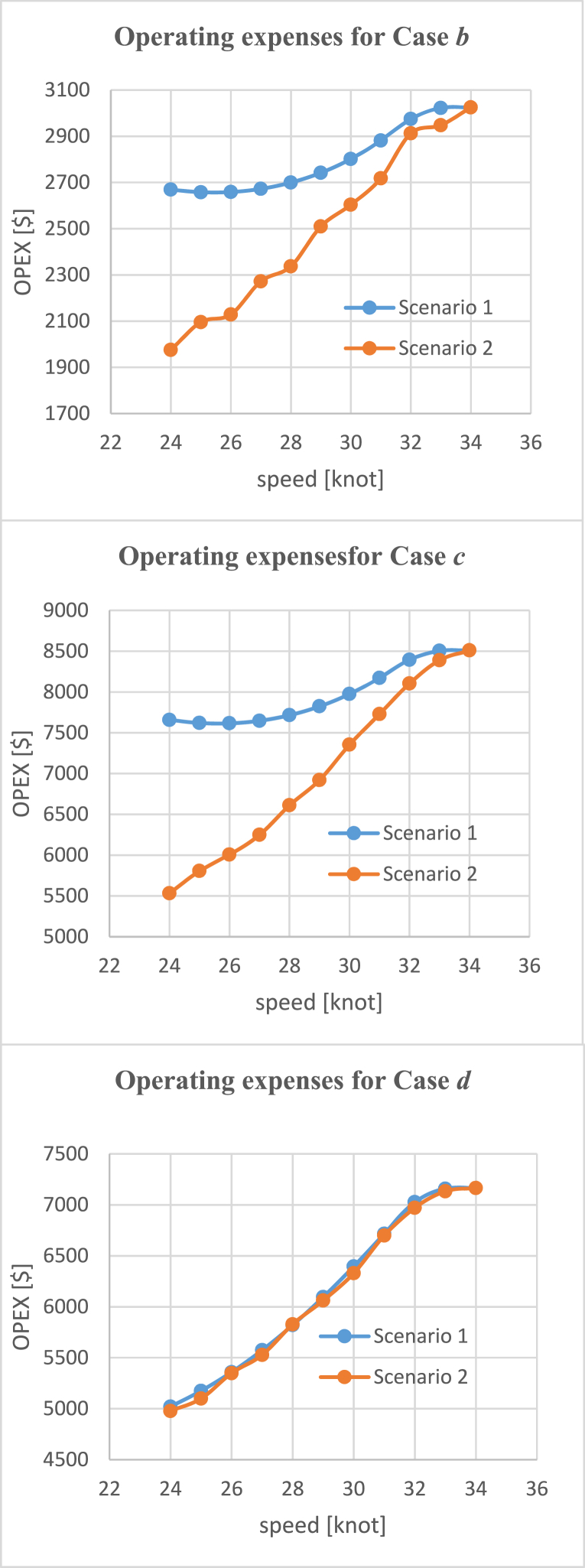
A comparison between hydrogen cases for the operating expenses.

Fig. 13 shows that the replacement of equipment in scenario 2 leads to a decrease in expenses compared with scenario 1. The difference in OPEX is noticeable for Case *b* and Case *c*. However, for Case *d*, which operates with a superconducting motor, the OPEX remains almost the same between the two scenarios. While scenario 2 allows for some cost savings due to the reduction in fuel cells, the difference in expenses is not notably significant for Case *d* compared to the other two cases. Specifically, the high-speed ferry requires 12 packs of fuel cells when operated at 34 knots, whereas it only needs seven packs of fuel cells when the maximum speed is reduced to 24 knots. This reduction in the number of fuel cell packs leads to a cost saving of about $500 per journey. A similar amount of money is also saved in other cases due to reduction of fuel cells besides some abatement cost in OPEX.

## 4 Conclusion

This study presents a comprehensive technical and cost analysis model for transitioning a high-speed ferry, specifically the Elsa Laula Renberg, from diesel to green hydrogen through a retrofit, achieving zero-emission operation. Both compressed gas and liquid hydrogen have been considered as potential fuels for the ferry. Different cases have been thoroughly investigated, analyzing the advantages and disadvantages of each approach. The study evaluates various propulsion systems, taking into account energy demand, fuel consumption, and overall cost-effectiveness.

At present, the CH_2_-fueled ferry stands out as the most economical option among all cases, despite having the highest energy demand and hydrogen consumption. However, we believe that the best option depends on the case study and could be different from one case study to another. For the case study under investigation, the operating expenses of the CH_2_ ferry are three times lower than that of LH_2_, and still slightly lower than the diesel option. However, the study suggests that a potential drop in LH_2_ cost with production at higher scales could reduce this gap considerably and make it competitive with both CH_2_ and diesel options in the future. The adoption of liquid hydrogen allows for the implementation of a superconducting motor in the propulsion system, leading to enhanced efficiency. Therefore, operating the ferry with LH_2_ and a superconducting motor not only represents the best technical option in terms of efficiency but also can potentially offer future economic viability, comparable to diesel and CH_2_ options. In terms of energy loss, CH_2_ and diesel ferries exhibit the highest energy losses (approximately 61,000 MJ), whereas LH_2_, especially when combined with a superconducting motor, experiences the least energy loss (approximately 41,000 MJ). The primary source of energy loss in diesel ferries is attributed to the engine, whereas in the hydrogen-based system, fuel cells constitute the major proportion of energy loss. To enhance efficiency and economic viability, it is beneficial to reduce the speed of high-speed ferries and optimize the onboard equipment, despite the resulting extension in journey duration. For example, the operating expenses and energy losses could decrease by around 50% for hydrogen ferries operating with the conventional propulsion system if the maximum speed of the ferry reduces to 24 knots from 34 knots. Without equipment optimization, changes in speed have a negligible effect on fuel consumption, and consequently, on the OPEX (fuel use) for hydrogen ferries equipped with a conventional motor system. These findings contribute to the ongoing efforts in sustainable maritime transportation and highlight the potential for zero-emission high-speed ferries powered by green hydrogen to become a viable and environmentally friendly alternative. The findings also highlight that the adoption of LH_2_ in this sector requires the development of LH_2_ production and its integration with innovative superconducting technology to make it economically viable.

## 5 Limitations and simplifications

In acknowledging the scope of this study, it is important to identify its limitations. Most of the restrictions were in the economic part due to confidentiality of data and its avalibility in literature. Therefore, the technical and cost analysis modelling has been performed based on the information, which could be acquired. Despite these challenges, the developed model remains adaptable, allowing for the inclusion of additional data to enhance the depth of analysis. For instance, the CAPEX of the superconducting motors and some other equipment, the maintenance cost, the crews’ salary, and the infrastructure cost of the refueling stations are examples of the parameters that could be added to the analysis in case related information could be obtained.

To conduct the analysis, some simplifications and assumptions were unavoidable. For example, the efficiency of the diesel engine is assumed constant throughout the whole operation, while in reality the efficiency of the engine fluctuates respect to the power load. To assess the impact of this assumption, a sensitivity test was done on the efficiency of the engine. Same assumption was considered for the gearbox as well.

## Data availability

There is no data appropriate to share.

## CRediT authorship contribution statement

**Masih Mojarrad:** Writing – original draft, Software, Methodology, Investigation, Formal analysis, Data curation, Conceptualization. **Rebecca Jayne Thorne:** Writing – original draft, Formal analysis, Conceptualization. **Kenneth Løvold Rødseth:** Writing – review & editing, Supervision, Conceptualization.

## Declaration of generative AI and AI-assisted technologies in the writing process

During the preparation of this work the authors used ChatGPT in order to improve readability and language. After using this tool/service, the authors reviewed and edited the content as needed and take full responsibility for the content of the publication.

## Declaration of competing interest

The authors declare that they have no known competing financial interests or personal relationships that could have appeared to influence the work reported in this paper.
